# Relationship between the characteristics of Japanese physicians involved in medical care for older adults and their approaches to treating older patients with multimorbidity

**DOI:** 10.1371/journal.pone.0302532

**Published:** 2024-06-12

**Authors:** Takuma Kimura, Kyoko Nomura, Masayoshi Hashimoto, Ken Shinmura

**Affiliations:** 1 Department of R&D Innovation for Home Care Medicine, Department of General Medicine, Tokyo Medical and Dental University School of Medicine, Tokyo, Japan; 2 Department of Environmental Health Science and Public Health, Akita University Graduate School of Medicine, Akita, Akita, Japan; 3 Department of General Internal Medicine, Hyogo Medical University School of Medicine, Nishinomiya, Hyogo, Japan; Faculty of Health Sciences - Universidade da Beira Interior, PORTUGAL

## Abstract

One countermeasure against the increasing prevalence of multimorbidity is the need to provide clinical education and training that considers the characteristics of physicians. We conducted a questionnaire survey to determine the relationship between physicians’ characteristics and their approach to treating older patients with multimorbidity. A total of 3300 geriatric specialists and primary care specialists in Japan were enrolled. A 4-point Likert scale was used to score the following items: difficult diseases (43 items), difficult patient backgrounds (14 items), important clinical factors (32 items), and important clinical management (32 items). Exploratory factor analysis was performed to examine the constructs in each of the scales Diseases, Backgrounds, Clinical Factors, and Clinical Management, and group comparisons by physician characteristics were conducted. A total of 778 respondents were included in the analysis. Six factors for Diseases, two factors for Patient Background, four factors for Clinical Factors, and two factors for Clinical Management were explored as patterns. Group comparison between mean scores for each factor and the characteristics of responding physicians showed statistically significant differences in at least one factor for all patterns in terms of years of experience as a physician (26 years or less, 27 years or more), the clinical setting (providing or not providing home medical care), and sex (male or female). Our results suggest a need for clinical education and training that takes into account not only physicians’ experience and clinical setting, but also their sex.

## Introduction

Multimorbidity, defined as the coexistence of multiple chronic diseases in a single patient, presents a significant challenge to health care systems worldwide [1, 2]. Multimorbidity leads to various outcomes, including increased polypharmacy, reduced quality of life (QOL), higher health care resource utilization, and mortality [3–7]. Efforts to enhance the quality of care have focused on clinical education and training, but their effectiveness has been limited [8–11]. To enhance education and training in multimorbidity care, it is crucial to understand the specific challenges faced by physicians and the key aspects of management that physicians prioritize.

In Europe and the United States, geriatricians and primary care physicians (e.g., general practitioners in the United Kingdom) play central roles in managing older patients [12–14]. Although it is unclear whether practice guidelines developed in Europe and the United States can be adapted to Japan’s health care system, geriatricians and primary care physicians in Japan are expected to be involved in the care of older patients with multimorbidity. We have previously examined the challenges faced by geriatricians and primary care physicians in Japan when treating older patients with multimorbidity, along with the emphasized management strategies, highlighting both similarities and differences between them [15]. However, the relationship remains unclear between physicians’ approaches to treating multimorbidity in older patients, as well as physician characteristics such as years of experience or the clinical setting in Japan. In Europe and the United States, various consultations for older patients with multimorbidity are provided in clinics, and the importance of generalists, such as general practitioners and geriatricians, as attending physicians in hospital wards has been highlighted. However, the relationship between the physician’s sex and experience as well as their approaches to treating multimorbidity in older patients is unclear [16, 17]. Understanding physicians’ characteristics is vital for tailoring strategies and clinical education with respect to multimorbidity.

Hence, we conducted a survey to elucidate the relationship between the characteristics of geriatricians and primary care physicians and their approach to treating older patients with multimorbidity.

## Methods

### Study population

Between June and July 2022, an unregistered postal survey was conducted among 3300 participants, including 1650, the total number of geriatric specialists (G specialists) from the Japan Geriatric Society and 1650 primary care specialists (PC specialists) randomly selected from among 1091, the total number of family medicine specialists and 5435, the total number of primary care physicians certified by the Japanese Primary Care Association (JPCA).

### Questionnaire

The questionnaire comprised two parts: respondents’ practice approach to multimorbidity and characteristics of the respondents. The practice model for treating older patients with multimorbidity was constructed based on previous studies, addressing difficult diseases/backgrounds and important clinical factors/clinical management among physicians [12–14, 16, 18–20]. The questionnaire was labeled "G" for G specialists and "P" for JPCA specialists.

For questions regarding their approach to treating older patients with multimorbidity, participants rated difficulties in treating diseases (43 items, Appendix 1 in S1 Appendix), patient backgrounds (14 items, Appendix 2 in S1 Appendix), important clinical factors (32 items, Appendix 3 in S1 Appendix), and important clinical management strategies (19 items, Appendix 4 in S1 Appendix) using a Likert scale from 1 ("not at all") to 4 ("very much"). Respondents also indicated their views regarding the difficulty in treating multimorbidity in older patients and the importance of general practice guidelines, also using a Likert scale.

As for respondents’ characteristics, participants provided information on their sex, age, years and months of experience as a physician, type of facility, clinical setting, municipality population size, and frequency of treating patients in specific age groups (65–75, 75–90, ≥90 years). They also disclosed their qualifications.

### Ethical considerations

This study received approval from the Ethical Review Committee of the Maruki Memorial Medical and Social Welfare Center, the first author’s previous institution (No. 37). The questionnaire cover page outlined the survey’s purpose, privacy protection, and provided contact information. Consent was inferred upon participant completion of the questionnaire.

### Statistical analysis

Regarding treating older patients with multimorbidity, a 4-point Likert scale was used to assess items in the categories of "Diseases," "Backgrounds," "Clinical Factors," and "Clinical Management,” with 1 denoting "not at all" and 4 indicating "very much." Mean and standard deviation were calculated. Respondents indicating "sometimes" or "often" were grouped together as "yes" in terms of having difficulty treating older patients with multimorbidity and the importance of referring to general practice guidelines; those who responded "not often" or "never" were grouped as "no."

Backgrounds of respondents were categorized into a "clinic group" (those working in a clinic with or without beds) or "hospital group" (those working in a hospital with <200 or >200 beds or university hospital). Population size was divided into "under 100,000" and "over 100,000." The frequency of treating certain age groups (65–75, 75–90, ≥90 years) was classified as "low frequency" (never or not often) or "high frequency" (sometimes or often).

Next, we explored constructs in the approach to treating older patients with multimorbidity. The items in the Diseases, Backgrounds, Clinical Factors, and Clinical Management scales were considered to be very diverse and interrelated; we considered that it would be useful to explore factors that commonly influence each item by examining the correlation between them. We reconstructed the scale at the smallest unit and then clarified the relationships with physicians’ characteristics. We conducted exploratory factor analysis using the maximum likelihood method and promax rotation to examine the constructs in each of the scales Diseases, Backgrounds, Clinical Factors, and Clinical Management in the approach to treating older patients with multimorbidity. First, an initial analysis was conducted using the number of factors with an eigenvalue of 1 in a scree plot; factor loadings greater than 0.40 were considered meaningful and used as a criterion for item selection [21]. Variables with factor loadings less than 0.4 or those included in two or more factors were then deleted [21]. The second and third rounds of analysis were conducted, adopting the factors that could be interpreted as the final model: "Disease patterns", "Background patterns, " "Clinical factor patterns", and "Clinical management patterns". Cronbach’s alpha coefficients were calculated for each scale overall and for each factor to verify internal consistency.

As a final analysis, group comparison of mean scores for each factor in each scale and characteristics of respondents was conducted. The mean scores for each of the factors Diseases, Backgrounds, Clinical Factors, Clinical Management were analyzed according to the following groups: sex, years of physician experience (below median, above median), practice facility (clinic, hospital), clinical setting (ward practice, home medical care, practice in a nursing home), municipality population (under 100,000, over 100,000), frequency of practice by age group (low-frequency group, high-frequency group), and general practice guidelines (emphasis, non-emphasis). Student *t*-tests were conducted for comparison, using SAS version 9.3 (SAS Institute Inc. Cary, NC, USA).

## Results

A total of 836 questionnaires were received (25.3% response rate), with 439 from G specialists (26.6% response rate) and 397 from JPCA (PC) specialists (24.06% response rate). In group G, four respondents either indicated that they lacked geriatric specialization or left their response blank, even though they were eligible for geriatric specialty certification. Additionally, in the PC group, 11 respondents either did not have family medicine certification or primary care certification or left their response blank, despite being eligible for family medicine or primary care certification. Thus, questionnaires from 15 respondents were excluded. To focus on physicians reporting difficulty in treating multimorbidity among older patients with a specific disease, we excluded 14 respondents who answered "not at all" to the question of whether they have difficulty treating older patients with multimorbidity and 29 questionnaires with responses missing for all items on treating difficult diseases. Finally, 778 cases were included in the analysis.

As shown in Table 1, in total, 629 (81.7%) physicians were men and 141 (18.3%) were women, with mean age 53.5±12.1 years. The mean length of career as a physician was 27.8±11.9 years, with a median of 27 years. As for age, 368 respondents were 26 years old or younger and 401 were 27 years old or older. In terms of facilities, 238 (32.9%) respondents worked in a clinic and 485 (67.1%) in a hospital; 734 (94.3%) respondents had an outpatient practice, 300 (38.6%) worked in home medical care, 215 (27.6%) practiced in nursing homes, and 413 (53.1%) had a ward practice. Regarding the population size of the municipalities where physicians treated patients, 207 (27.2%) worked in municipalities with fewer than 100,000 residents and 554 (72.8%) were from municipalities with more than 100,000. Regarding the frequency of treating patients aged 65 to 75 years, 4 (0.5%) physicians reported low frequency and 771 (99.5%) reported high frequency; for patients aged 75 to 90 years, three (0.4%) physicians reported low frequency and 774 (99.6%) reported high frequency; and for treating patients over 90 years old, 39 (5.0%) physicians reported low frequency and 739 (95.0%) reported high frequency. Regarding qualifications, 505 (64.9%) physicians stated that they were a fellow of the Japanese Society of Internal Medicine, 396 (50.9%) were board-certified members of the Japanese Society of Internal Medicine, 415 (53.3%) were geriatric specialists, 369 (47.4%) were primary care-certified physicians, 113 (14.5%) were family medicine specialists, and 34 (4.4%) physicians were specialists in home medical care.

**Table 1:** Characteristics of respondents (N = 778).

	n or median	(%) or 1st quartile,3rd quartile
Sex		
Male	629	(81.7)
Female	141	(18.3)
Age, years	53.0	44.0,62.0
Career as a physician (years)	27.0	18.0,27.0
Facility		
Clinic	238	(32.0)
Hospital	485	(65.2)
Care facility	13	(0.1)
Other		
Clinical setting		
Outpatient practice	734	(94.3)
Home medical care	300	(38.6)
Practice at nursing home	215	(27.6)
Ward practice	413	(53.1)
Population size of the municipality		
Over 100,000	554	(72.8)
Under 100,000	207	(27.2)
Treating patients aged 65 to 75 years		
High-frequency group	771	(99.5)
Low-frequency group	4	(0.5)
Treating patients aged 75 to 90 years		
High-frequency group	774	(99.6)
Low-frequency group	3	(0.4)
Treating patients aged 90 years or older		
High-frequency group	739	(95.0)
Low-frequency group	39	(5.0)
Qualifications		
Fellow of the Japanese Society of Internal Medicine	505	(64.9)
Board Certified Member, Japanese Society of Internal Medicine	396	(50.9)
Geriatric specialist	415	(53.3)
Primary care certified physician	369	(47.4)
Family medicine specialist	113	(14.5)
Home medical care specialist	34	(4.4)

### Descriptive statistics

Tables 2 to 5 show descriptive statistics for difficult diseases, difficult backgrounds, clinical factors, and clinical management, respectively. As for referring to general practice guidelines, 33 physicians reported "sometimes," 582 answered "often," 139 stated "not often," and seven physicians said they "never" referred to the guidelines. Thus, 615 respondents were categorized in the "yes" group and 146 were in the "no" group.

**Table 2:** Descriptive statistics of difficult diseases.

			Not at all difficult		Not very difficult		Quite difficult		Very difficult	
				%		%		%		%
Difficult diseases										
1) Congestive heart failure	N =	776	33	4.24	179	23.01	427	54.88	137	17.61
2) Hypertension	N =	777	203	26.09	444	57.07	125	16.07	5	0.64
3) Atrial fibrillation	N =	776	64	8.23	305	39.2	370	47.56	37	4.76
4) Ischemic heart disease	N =	774	44	5.66	255	32.78	395	50.77	80	10.28
5) Peripheral arterial disease	N =	777	31	3.98	190	24.42	409	52.57	147	18.89
6) Diabetes mellitus (severe)	N =	777	55	7.07	222	28.53	404	51.93	96	12.34
7) Diabetes mellitus (mild)	N =	775	152	19.54	432	55.53	180	23.14	11	1.41
8) Dyslipidemia	N =	777	329	42.29	412	52.96	35	4.5	1	0.13
9) Gout/hyperuricemia	N =	777	302	38.82	428	55.01	45	5.78	2	0.26
10) Thyroid disease	N =	776	144	18.51	486	62.47	140	17.99	6	0.77
11) Chronic lung disease	N =	774	41	5.27	186	23.91	452	58.1	95	12.21
12) Chronic kidney disease (moderate-to-severe)	N =	777	34	4.37	110	14.14	364	46.79	269	34.58
13) Cerebrovascular disease (severe)	N =	777	102	13.11	350	44.99	286	36.76	39	5.01
14) Hemiplegia	N =	775	60	7.71	282	36.25	356	45.76	77	9.9
15) Neurological intractable disease	N =	773	22	2.83	77	9.9	294	37.79	380	48.84
16) Peptic ulcer	N =	776	136	17.48	413	53.08	210	26.99	17	2.19
17) Inflammatory bowel disease	N =	771	27	3.47	222	28.53	431	55.4	91	11.7
18) Constipation	N =	776	124	15.94	415	53.34	218	28.02	19	2.44
19) Collagen disease	N =	774	15	1.93	137	17.61	443	56.94	179	23.01
20) Liver disease (mild)	N =	775	48	6.17	371	47.69	320	41.13	36	4.63
21) Moderate to severe hepatic dysfunction	N =	774	13	1.67	111	14.27	387	49.74	263	33.8
22) Solid tumor	N =	775	68	8.74	322	41.39	280	35.99	105	13.5
23) Cancer metastasis/metastatic solid cancer	N =	774	12	1.54	127	16.32	322	41.39	313	40.23
24) Lymphoma	N =	772	8	1.03	100	12.85	332	42.67	332	42.67
25) Leukemia or true erythrocytosis	N =	771	5	0.64	72	9.25	253	32.52	441	56.68
26) Depression	N =	771	13	1.67	141	18.12	448	57.58	169	21.72
27) Dementia	N =	774	34	4.37	204	26.22	338	43.44	198	25.45
28) Sleep disorders	N =	772	46	5.91	340	43.7	350	44.99	36	4.63
29) Benign prostatic hyperplasia	N =	771	72	9.25	428	55.01	254	32.65	17	2.19
30) Vertigo	N =	772	46	5.91	383	49.23	329	42.29	14	1.8
31) Hearing loss	N =	773	32	4.11	284	36.5	336	43.19	121	15.55
32) Low back pain	N =	774	50	6.43	411	52.83	282	36.25	31	3.98
33) Epilepsy	N =	770	22	2.83	274	35.22	378	48.59	96	12.34
34) Spinal canal stenosis	N =	773	33	4.24	325	41.77	353	45.37	62	7.97
35) Osteoporosis	N =	774	112	14.4	462	59.38	190	24.42	10	1.29
36) Osteoarthritis	N =	771	59	7.58	417	53.6	270	34.7	25	3.21
37) Visual impairment	N =	771	17	2.19	251	32.26	400	51.41	103	13.24
38) Glaucoma	N =	771	33	4.24	281	36.12	334	42.93	123	15.81
39) Cataract	N =	771	95	12.21	438	56.3	178	22.88	60	7.71
40) Dental problems	N =	772	46	5.91	351	45.12	296	38.05	79	10.15
41) Bedsores	N =	771	22	2.83	187	24.04	386	49.61	176	22.62
42) Eczema/dermatitis	N =	772	66	8.48	401	51.54	283	36.38	22	2.83
43) AIDS	N =	744	18	2.31	92	11.83	200	25.71	434	55.78

Response patterns were based on a 4-point Likert scale from totally disagree = 1 to totally agree = 4.

**Table 3:** Descriptive statistics of difficult backgrounds.

			Not at all difficult		Not very difficult		Quite difficult		Very difficult	
				%		%		%		%
Difficult backgrounds										
1) Severe comorbidities	N =	772	6	0.77	48	6.17	342	43.96	376	48.33
2) Many social problems	N =	772	5	0.64	43	5.53	288	37.02	436	56.04
3) Many psychiatric/psychological problems	N =	771	3	0.39	42	5.4	316	40.62	410	52.7
4) Difficulty in setting the goals and outcomes for medical treatment	N =	771	6	0.77	83	10.67	400	51.41	282	36.25
5) Difficult communicating with patients	N =	771	9	1.16	79	10.15	361	46.4	322	41.39
6) Difficulty communicating with family members	N =	771	13	1.67	97	12.47	331	42.54	330	42.42
7)No key person is available	N =	772	7	0.9	106	13.62	343	44.09	316	40.62
8) Patient lives alone	N =	771	11	1.41	137	17.61	389	50	234	30.08
9) Difficult to identify the department/medical institution where the patient is receiving treatment at another clinic or hospital	N =	772	13	1.67	145	18.64	419	53.86	195	25.06
10) Difficulty collecting clinical information during outpatient visits to other clinics or hospitals	N =	772	11	1.41	134	17.22	420	53.98	207	26.61
11) Difficulty collecting clinical information when the patient is admitted to or discharged from another hospital	N =	772	8	1.03	184	23.65	412	52.96	168	21.59
12) Unable to follow general practice guidelines	N =	771	20	2.57	231	29.69	113	52.31	113	14.52
13) Difficulty in collaborating with specialists in organs/areas	N =	771	12	1.54	244	31.36	129	49.61	129	16.58
14) Differences of opinion on goal setting with the specialist in the organ/area	N =	768	9	1.16	207	26.61	388	49.87	164	21.08

Response patterns were based on a 4-point Likert scale from totally disagree = 1 to totally agree = 4.

**Table 4:** Descriptive statistics of clinical factors.

			Not at all important		Not very important		Quite important		Very important	
				%		%		%		%
Clinical factors										
1) Comorbidities in the primary disease	N =	770	0	0	15	1.93	511	65.68	244	31.36
2) Hearing loss	N =	771	7	0.9	261	33.55	437	56.17	66	8.48
3) Visual impairment	N =	772	4	0.51	217	27.89	468	60.15	83	10.67
4) Wheelchair ADLs	N =	771	7	0.9	126	16.2	493	63.37	145	18.64
5) Bedridden ADLs	N =	769	5	0.64	56	7.2	349	44.86	359	46.14
6) Cognitive impairment	N =	772	1	0.13	29	3.73	369	47.43	373	47.94
7) Depression	N =	771	2	0.26	62	7.97	499	64.14	208	26.74
8) Low nutrition	N =	771	0	0	27	3.47	376	48.33	368	47.3
9) Psychiatric complications	N =	772	0	0	38	4.88	443	56.94	291	37.4
10) Urinary incontinence	N =	773	12	1.54	213	27.38	490	62.98	58	7.46
11) History of falls	N =	773	3	0.39	42	5.4	490	62.98	238	30.59
12) Certified as requiring long-term care insurance	N =	773	5	0.64	97	12.47	452	58.1	219	28.15
13) Poor adherence to medications	N =	774	0	0	13	1.67	340	43.7	421	54.11
14) Polypharmacy	N =	773	1	0.13	26	3.34	395	50.77	351	45.12
15) Burdened by waiting time for outpatient department	N =	772	5	0.64	162	20.82	518	66.58	87	11.18
16) Burdened by visits to the doctor and outpatient department	N =	773	1	0.13	49	6.3	527	67.74	196	25.19
17) Exercise therapy decreases QOL	N =	771	17	2.19	183	23.52	487	62.6	84	10.8
18) Dietary therapy decreases QOL	N =	772	9	1.16	130	16.71	502	64.52	131	16.84
19) Medication decreases QOL	N =	772	1	0.13	72	9.25	488	62.72	211	27.12
20) Burden of treatment	N =	773	2	0.26	22	2.83	394	50.64	355	45.63
21) Limited support of daily living	N =	774	0	0	6	0.77	395	50.77	373	47.94
22) Decreased ability for the patient to be cared for at home	N =	774	0	0	3	0.39	329	42.29	442	56.81
23) Repeated emergency room visits	N =	773	0	0	31	3.98	337	43.32	405	52.06
24) Repeated hospitalization and discharge	N =	773	0	0	20	2.57	330	42.42	423	54.37
25) Financial problems	N =	773	0	0	38	4.88	400	51.41	335	43.06
26) Living alone	N =	774	2	0.26	46	5.91	401	51.54	325	41.77
27) Absence of a physician who coordinates and decides the overall medical policy	N =	774	5	0.64	75	9.64	390	50.13	304	39.07
28) Number of medical institutions attended	N =	774	5	0.64	123	15.81	538	69.15	108	13.88
29) Number of coexisting diseases	N =	773	6	0.77	85	10.93	519	66.71	163	20.95
30) Age	N =	774	7	0.9	110	14.14	475	61.05	182	23.39
31) Estimated life expectancy	N =	774	3	0.39	33	4.24	349	44.86	389	50
32) Frailty	N =	773	1	0.13	21	2.7	447	57.46	304	39.07

Response patterns were based on a 4-point Likert scale from totally disagree = 1 to totally agree = 4.

ADLs, activities of daily living; QOL, quality of life.

**Table 5:** Descriptive statistics of clinical management.

			Not at all important		Not very important		Quite important		Very important	
				%		%		%		%
Clinical management										
1) Refer to practice guidelines with older adults in mind	N =	776	3	0.39	74	9.51	525	67.48	174	22.37
2) Review evidence on important outcomes	N =	775	0	0	93	12	561	72.11	121	15.55
3) Review indications for drug therapy for primary prevention	N =	776	5	0.64	123	15.8	492	63.24	156	20.05
4) Review indications for drug therapy for secondary prevention	N =	775	0	0	51	6.56	527	67.74	197	25.32
5) Re-evaluate drugs	N =	776	0	0	27	3.47	461	59.25	288	37.02
6) Reassess treatment strategy	N =	776	0	0	16	2.06	467	60.03	293	37.66
7) Evaluate patient treatment burden	N =	776	1	0.13	21	2.7	434	55.78	319	41
8) Listen to the patient’s wishes and values	N =	776	0	0	5	0.64	272	34.96	499	64.14
9) Listen to the opinions of the family	N =	776	0	0	12	1.54	353	45.37	411	52.83
10) Ask the opinions of other professionals	N =	776	0	0	34	4.37	459	59	283	36.38
11) Ask for the opinions of people the patient trusts	N =	775	7	0.9	115	14.8	474	60.93	179	23.01
12) Present options to the patient regarding treatment goals	N =	776	0	0	20	2.57	427	54.88	329	42.29
13) Present treatment priorities to the patient	N =	776	1	0.13	20	2.57	419	53.86	336	43.19
14) Consolidating physicians who will treat patients	N =	776	5	0.64	129	16.6	518	66.58	124	15.94
15) Identify a physician who will determine the overall treatment plan	N =	776	3	0.39	82	10.5	482	61.95	209	26.86
16) Examine the intervals between visits to an organ/area specialist	N =	776	8	1.03	148	19	547	70.31	73	9.38
17) Clarify the role of the organ/area specialist	N =	776	2	0.26	95	12.2	534	68.64	145	18.64
18) Use of long-term care insurance services	N =	776	0	0	14	1.8	300	38.56	462	59.38
19) Multidisciplinary intervention	N =	776	1	0.13	23	2.96	344	44.22	408	52.44

Response patterns were based on a 4-point Likert scale from totally disagree = 1 to totally agree = 4.

ADLs, activities of daily living; QOL, quality of life.

### The constructs in the approach to treating older patients with multimorbidity

Six factors were explored regarding "Disease patterns" and the overall Cronbach’s alpha coefficient was 0.937 (Table 6). Factor 1 encompassed seven items including congestive heart failure, diabetes mellitus (with complications), and chronic kidney disease. This was termed "cardiovascular disease with diabetes mellitus" (vascular), indicating patients with advanced atherosclerosis, a history of smoking, and diabetes as underlying conditions. Factor 2 included five items involving lifestyle-related diseases like hypertension, diabetes mellitus (without complications), dyslipidemia, and thyroid disease. This was labeled "outpatient internal medicine diseases" (outpatient) to indicate continual practice in a general internal medicine outpatient clinic. Factor 3 comprised three items covering cerebrovascular diseases and neurological intractable diseases, labeled "neurological disease" (neurological). Factor 4 comprised five items including solid tumors, cancer metastasis, lymphoma, leukemia, and AIDS. This was designated "malignant tumor, hematologic diseases, and AIDS" (malignancy). Factor 5 consisted of 13 items encompassing dementia, benign prostatic hyperplasia, osteoarthritis, and others. This factor included pathologies of various organs associated with aging and was denoted "geriatric syndrome" (geriatric). Factor 6 included four items related to visual impairment, cataracts, and dental problems and was termed "ophthalmologic and dental" (ophthal).

**Table 6:** Disease patterns, 6 factors, 37 items (Cronbach’s alpha coefficient = 0.937).

	Factor 1	Factor 2	Factor 3	Factor 4	Factor 5	Factor 6
Factor 1: 7 items "cardiovascular disease with diabetes mellitus" (Cronbach’s alpha coefficient = 0.888)						
1) Congestive heart failure	0.05713	0.81294	-0.11215	-0.026	-0.00429	-0.09153
3) Atrial fibrillation	-0.05868	0.69383	0.01111	0.12817	-0.06089	0.08638
4) Ischemic heart disease	-0.10664	0.84832	0.14092	0.02473	-0.01339	0.01774
5) Peripheral arterial disease	-0.05092	0.81398	0.11887	-0.07191	-0.01583	-0.00015
6) Diabetes mellitus (severe)	0.0655	0.63127	-0.04467	0.07763	0.01156	0.00991
11) Chronic lung disease	0.18853	0.5311	-0.09053	-0.00405	0.05966	0.10632
12) Chronic kidney disease (moderate-to-severe)	0.0992	0.54822	-0.14935	-0.0504	0.14331	0.04659
Factor 2: 5 items "Outpatient internal medicine diseases" (Cronbach’s alpha coefficient = 0.854)						
2) Hypertension	0.12707	0.30526	-0.0003	0.47406	-0.05685	-0.0099
7) Diabetes mellitus (mild)	0.1141	0.2209	-0.05952	0.46791	0.02178	0.08187
8) Dyslipidemia	-0.02996	-0.08965	-0.03009	0.97852	0.06447	-0.00006
9) Gout/hyperuricemia	0.0341	-0.02262	-0.01682	0.88224	0.00124	-0.00419
10) Thyroid disease	-0.04246	0.20983	0.14634	0.44099	0.08942	0.00623
Factor 3: 3 items "Neurological disease" (Cronbach’s alpha coefficient = 0.768)						
13) Cerebrovascular disease(severe)	0.04374	0.1351	0.08247	0.23538	-0.10772	0.59339
14) Hemiplegia	0.03159	0.1003	0.05344	0.06231	-0.02206	0.78248
15) Neurological intractable disease	0.01215	0.02743	0.03032	-0.13205	0.21351	0.57788
Factor 4: 5 items "Malignant tumor, hepatic dysfunction, and AIDS" (Cronbach’s alpha coefficient = 0.813)						
22) Solid tumor	0.08514	-0.01348	0.05906	0.23	0.45099	0.06577
23) Cancer metastasis/metastatic solid cancer	0.21912	0.06076	-0.10945	-0.00373	0.62759	0.06666
24) Lymphoma	-0.00639	-0.04521	-0.01081	0.08602	0.92808	-0.01703
25) Leukemia or true erythrocytosis	-0.10639	0.02093	0.07784	-0.00193	0.82835	0.04211
43) AIDS	-0.11263	0.06506	0.23935	-0.06895	0.47617	-0.03855
Factor 5: 13 items "Geriatric syndrome" (Cronbach’s alpha coefficient = 0.899)						
18) Constipation	0.59464	0.0448	-0.10155	0.19871	-0.07789	-0.02814
26) Depression	0.48345	0.02316	0.03924	-0.05988	0.22822	0.09305
27) Dementia	0.52583	0.02286	-0.2279	-0.00577	0.00494	0.33859
28) Sleep disorders	0.72757	-0.01521	-0.08833	0.02499	-0.03008	0.03191
29) Benign prostatic hyperplasia	0.54119	0.07718	0.13498	0.14635	0.10412	-0.19508
30) Vertigo	0.65589	0.01385	0.01314	0.04546	-0.1023	0.08447
31) Hearing loss	0.48166	-0.06379	0.2364	-0.0823	-0.00276	0.12749
32) Low back pain	0.73951	-0.02798	0.185	-0.02872	-0.07055	-0.03054
34) Spinal canal stenosis	0.55641	-0.01478	0.34236	-0.0675	0.01791	0.04761
35) Osteoporosis	0.46582	0.06181	0.20009	0.20902	-0.04873	-0.03559
36) Osteoarthritis	0.55802	-0.00158	0.29763	0.06335	-0.02251	-0.05336
41) Bedsores	0.57398	0.13463	-0.02934	-0.13347	0.14621	0.03057
42) Eczema/dermatitis	0.43851	0.06465	0.2677	0.12027	0.04129	-0.14051
Factor 6: 4 items "Ophthalmologic and dental problems" (Cronbach’s alpha coefficient = 0.825)						
37) Visual impairment	0.21662	-0.03725	0.55858	-0.10821	0.0457	0.17849
38) Glaucoma	-0.01496	0.02478	0.74406	-0.02871	0.14267	-0.06313
39) Cataract	-0.02574	-0.06913	0.83402	0.09898	-0.00223	0.07246
40) Dental problems	0.1731	0.00687	0.63562	-0.00163	-0.02936	-0.02124

Numbers enclosed in squares indicate selected factors with factor loadings all ≥0.40.

Two factors were explored regarding "Background patterns" and the overall Cronbach’s alpha was 0.881 (Table 7). The first factor comprised six items including social and psychological issues, named "complex background” (complex). The second factor involved five items, such as challenges in collecting clinical information and collaborating with specialists of organs or areas, denoted "difficulty in collecting clinical information and cooperation" (lack of information and cooperation).

**Table 7:** Background patterns, 2 factors, 11 items (Cronbach’s alpha coefficient = 0.881).

	Factor 1	Factor 2
Factor 1: 6 items "Complex background" (Cronbach’s alpha coefficient = 0.850)			
1) Severe comorbidities	-0.01313	0.62105
2) Many social problems	-0.05422	0.83981
3) Many psychiatric/psychological problems	-0.01682	0.79014
4) Difficulty in setting the goals and outcomes for medical treatment	0.14084	0.59819
5) Difficult communicating with patients	0.18482	0.58474
6) Difficult to communicate with family members	0.19854	0.51448
Factor 2: 5 items "Difficulty in collecting clinical information and cooperation" (Cronbach’s alpha coefficient = 0.881)			
9) Difficulty identifying the department/medical institution where the patient is receiving treatment at another clinics or hospital	0.79526	0.04512
10) Difficulty in collecting clinical information during outpatient visits to other clinics or hospitals	0.934	-0.02343
11) Difficulty in collecting clinical information when the patient is admitted to or discharged from another hospital	0.85925	-0.0269
13) Difficulty in collaborating with specialists in organs/areas	0.47303	0.12667
14) Differences of opinion on goal setting with the specialist in the organ/area	0.45858	0.10867

Numbers enclosed in squares indicate selected factors with factor loadings all ≥0.40.

Four factors were explored for "Clinical factor patterns" and the overall Cronbach’s alpha coefficient was 0.844 (Table 8). Factor 1 encompassed six items regarding bedridden activities of daily living (ADL), nutrition, and psychiatric complications, termed "low ADL/nutrition, and psychiatric problems" (low ADL and psychiatry). Factor 2 comprised three items related to therapies affecting QOL, termed "treatment-induced QOL decrease" (QOL decrease). Factor 3 comprised three items concerning treatment burden and limited daily life support, denoted "treatment burden and difficulties in daily life" (burden). Factor 4 comprised three items including age, number of medical institutions, and coexisting diseases, termed "age and multiple medical conditions" (multiple medical).

**Table 8:** Clinical factor patterns, 4 factors, 15 items (Cronbach’s alpha coefficient = 0.844).

	Factor 1	Factor 2	Factor 3	Factor 4
Factor 1: 6 items "Low ADL/nutrition, and psychiatric problems" (Cronbach’s alpha coefficient = 0.814)				
4) Wheelchair ADLs	0.63984	0.11854	0.0946	0.14855
5) Bedridden ADLs	0.72013	0.04389	0.09763	0.09432
6) Cognitive impairment	0.70974	0.05519	0.1889	0.09349
7) Depression	0.52107	0.11092	0.15951	0.21638
8) Low nutrition	0.46158	0.12779	0.1798	0.16954
9) Psychiatric complications	0.55947	0.12048	0.17157	0.21718
Factor 2: 3 items "Treatment-induced QOL decrease" (Cronbach’s alpha coefficient = 0.795)				
17) Exercise therapy decreases QOL	0.10054	0.74609	0.09781	0.08624
18) Dietary therapy decreases QOL	0.14122	0.85191	0.15437	0.0697
19) Medication decreases QOL	0.12305	0.57633	0.26538	0.14512
Factor 3: 3 items "Treatment burden and difficulties in daily life" (Cronbach’s alpha coefficient = 0.773)				
20) Burden of treatment	0.13068	0.2626	0.48963	0.08734
21) Limited support of daily living	0.24887	0.16056	0.84405	0.14664
22) Decreased ability to home care for the patient	0.2855	0.13439	0.70512	0.11007
Factor 4: 3 items "Age and multiple medical conditions" (Cronbach’s alpha coefficient = 0.635)				
28) Number of medical institutions attended	0.13212	0.13864	0.13994	0.48709
29) Number of coexisting disease	0.18344	0.05621	0.09471	0.7672
30) Age	0.18539	0.04797	0.0338	0.47398

ADLs, activities of daily living; QOL, quality of life.

Numbers enclosed in squares indicate selected factors with factor loadings all ≥0.40.

Two factors were explored regarding "Clinical management patterns" and the overall Cronbach’s alpha coefficient was 0.78 (Table 9). The first factor included three items on re-evaluating treatment, named "re-establishment of treatment goals" (re-establishment). The second factor encompassed five items including patient preferences and collaboration among professions, termed "confirmation of clinical preference and collaboration among multiple professions" (confirmation and collaboration).

**Table 9:** Clinical management patterns, 2 factors, 8 items (Cronbach’s alpha coefficient = 0.780).

	Factor 1	Factor 2
Factor 1: 3 items "Re-establishment of treatment goals" (Cronbach’s alpha coefficient = 0.748)		
3) Review indications for drug therapy for primary prevention	0.01198	0.51664
5) Re-evaluate drugs	-0.04613	0.89006
6) Reassess treatment strategy	0.06779	0.73151
Factor 2: 5 items "Confirmation of clinical preference and collaboration among multiple professions" (Cronbach’s alpha coefficient = 0.758)		
8) Listen to the patient’s wishes and values	0.56976	0.07114
9) Listen to the opinions of the family	0.61005	-0.01573
10) Ask the opinions of other professionals	0.54193	0.16192
18) Use of long-term care insurance services	0.65914	-0.08273
19) Multidisciplinary intervention	0.65688	0.02155

Numbers enclosed in squares indicate selected factors with factor loadings all ≥0.40.

### Comparison of mean scores for each factor of each scale and characteristics of respondents

The S1 Table includes an overall results for the comparison of mean scores for each factor of each scale and characteristics of respondents. S1 Fig shows a box-plot diagram of factors with statistically significant differences in the group comparison.

For sex, in "Disease patterns", men scored higher in "malignancy." For "Background patterns," women scored higher in "lack of information and cooperation." In "Clinical factor patterns”, women had higher scores in "QOL decrease" and "burden." In "Clinical management patterns," women scored higher in "re-establishment" and "confirmation and collaboration."

Regarding years of experience as a physician, in "Disease patterns," older physicians scored higher in "malignancy" and "ophthal." For "Background patterns," younger physicians scored higher in "complex" and "lack of information and cooperation." In "Clinical factor patterns," younger physicians had higher scores for "burden." In "Clinical management patterns," younger physicians scored higher in "re-establishment" and "confirmation and collaboration."

As for facilities, hospital physicians scored higher in "complex" than clinic physicians in the same category. In terms of clinical setting, in "Disease patterns," the group with home medical care "not provided" scored higher in "neurological," "malignancy," and "geriatric. "For "complex" in "Background patterns," the group reporting ward practice "provided" scored higher than "not provided" and the group reporting home medical care "not provided" scored higher than "provided". In "Clinical factor patterns, " the group with home medical care "not provided" had higher scores in "burden." In "Clinical management patterns," the group reporting home medical care "provided" scored higher in "confirmation and collaboration." No differences were found between the "not provided" and "provided" groups in nursing homes.

Regarding population size, the mean value of "geriatric" in "Disease patterns" was significantly higher in the "over 100,000" group. In the frequency of practice among patients aged 65–74 years and 75–90 years, there were no significant differences between the low-frequency group and high-frequency group. As for the frequency of practice among patients aged 90 years or older, mean values for the high-frequency group were significantly higher for "burden" in "Clinical factor patterns" and "confirmation and collaboration" in "Clinical management patterns." For general practice guidelines, the non-emphasis group was significantly higher for "burden" in "Clinical factor patterns. "

## Discussion

In this study, we examined the relationship between difficult disease/patient background, important clinical factors/management and physician characteristics when treating older patients with multimorbidity.

Regarding sex, men perceived more difficulty in treating "malignancy "in "Disease patterns" than women. Women reported greater difficulty than men in treating "geriatric" in "Disease patterns" and "lack of information and cooperation" in "Background patterns." These may be related to the fact that women are more critical in their self-evaluation than men [22]. Additionally, women prioritized "QOL decrease" and "burden" in "Clinical factors pattern," along with "re-establishment" and "confirmation and collaboration" in "Clinical management patterns," more so than men. A study comparing male and female physicians revealed lower 30-day mortality and re-admission rates in older patients (65 years and older) treated by female physicians for internal medicine-related conditions [23]. Further research on the distinct approaches of female and male physicians in managing older patients with multimorbidity is encouraged.

Physicians with less experience prioritized "burden" in "Clinical factor patterns" and "re-establishment" and "confirmation and collaboration" in "Clinical management patterns" more frequently than their counterparts with greater experience. These aspects align with guidelines such as those of the National Institute for Health and Care Excellence, but variations in educational exposure might also play a role. In terms of physician experience and practice style, it was observed that more experienced physicians tended to show lower adherence to diagnostic criteria [24]. Whereas this is not specific to multimorbidity practice, it underscores the importance of ongoing education for seasoned physicians regarding the focal points of multimorbidity care.

Concerning facilities, "complex" in "Background patterns" was a greater concern among physicians in hospitals than those in clinics. It has been pointed out that complex backgrounds are one of the central issues in multimorbidity practice [25]. To address this complexity, the medical treatment approach should consider the type of facility, whether a clinic or a hospital.

Concerning the clinical setting, those involved in ward practice found "complex" factors in "Background patterns" more challenging than those not in a ward practice. Variations in disease patterns are linked to differences in re-admission and in-hospital mortality rates [26]. Further research into medical treatment content and styles for multimorbidity in older patients is advisable.

Physicians involved in home medical care were less likely to experience difficulties in managing "neurological," "malignancy," and "geriatric" in "Disease patterns." In this study, "geriatric" includes dementia. It has been reported that the coexistence of dementia in diabetes mellitus is a factor that makes it difficult to introduce home medical care [27]. In home health care, patients with severe dementia and diabetes are difficult to refer, and this may have affected the results. This may need to be discussed when treating older patients with multimorbidity, as well as the presence or absence of medical procedures such as ventilators [28]. Regarding "neurological," it is clear that ventilator-dependent patients, especially those with intractable neurological diseases, require specialized attention [28].

"Complex" in "Background patterns" was found to be less difficult for the group involved in home medical care. Home care patients are considered more complex than ward patients owing to the number of comorbidities and comorbid cognitive dysfunction [29, 30]. Clarification is needed regarding the background of visiting physicians who do not consider complex backgrounds to be difficult to treat. It is also possible that physicians who do not consider complex backgrounds difficult to treat may be providing home medical care. Additionally, "burden" in "Clinical factor patterns" was found to be more important when home medical care was provided. Patients with multimorbidity were reported to perceive a treatment burden in terms of medication and impact on daily life (including involvement of caregivers) [31]. It is possible that physicians who provide home medical care are more likely to perceive the impact on their daily lives and recognize the burden of treatment. Furthermore, physicians providing home medical care emphasized "confirmation and collaboration" in "Clinical management patterns" more than their counterparts in other clinical settings. Whereas multidisciplinary collaboration has limited effects on managing multimorbidity, there are reports suggesting that home rehabilitation therapy led by professionals can improve functional outcomes for patients with multimorbidity; this warrants further research [32, 33].

We observed that the group that did not prioritize general practice guidelines placed greater emphasis on "burden" in "Clinical factor patterns." These results suggest that general practice guidelines should be emphasized in medical care, but it is also necessary to educate physicians about the importance of "burden" as a patient outcome.

Limitations of this study include the following. First, the present responses may differ from actual practice, and we were unable to comprehensively examine the practice of multimorbidity in older patients. Thus, there may be unmeasured items and potential confounders. Furthermore, this study was a cross-sectional survey, and we only examined inter-relationships at the time of the survey. In the future, qualitative methods such as interviews and clinical scenarios can be used to examine multimorbidity treatment in more detail.

Second, the Cronbach’s alpha coefficients for the four patterns identified in exploratory factor analysis were above a certain level, suggesting that internal consistency was ensured, to some extent. The validity of this information is limited, although it is interesting to infer the thought process of physicians in actual clinical practice regarding the treatment of older patients with multimorbidity. It is important to note that the patterns of diseases in multimorbidity can substantially impact patient outcomes, affecting ADL and health-related QOL [4, 5, 34, 35]. Therefore, physicians should consider the identified patterns ("Disease patterns," "Background patterns," "Clinical factor patterns," and "Clinical management patterns"). Further validation in confirmatory factor analysis and other methods when managing multimorbidity should be done. As a next step in clinical education and training, a guide for treating "vascular" in "Disease patterns" in a clinic visit as a place of care, or a guide for treating "ambulatory internal medicine diseases" in an outpatient clinic setting could be considered [8–10].

Third, whether the results of this study can be generalized is debatable, especially because the response rate was 25.3%. However, the surveyed physicians, including geriatric and randomly selected primary care specialists, have a certain representativeness of physicians engaged in the medical management of older people in Japan. It is therefore possible to use the present results as basic data regarding the treatment of older patients with multimorbidity and the characteristics of physicians. However, the ratio of geriatricians and primary care specialists to the total number of physicians in Japan is quite low, and there are limitations in predicting overall trends among physicians based on the results of this study. Considering clinical education such as intensive lectures to improve knowledge levels may be warranted, for example, for "geriatric syndromes" or workshop-style training programs for problem-solving in the overall approach to multimorbidity, as well as conducting empirical studies [8–11].

Fourth, it is possible that the respondents in this study represent a group that had available time to participate in the survey and their opinions may not reflect those of geriatricians and general practitioners who are overworked. Moreover, with a median age of 53 years, the responses may not reflect the opinions of relatively young physicians.

In conclusion, we found that among geriatricians and primary care physicians, the approach to treating older patients with multimorbidity was related to the years of experience and clinical setting as well as the physician’s sex. The present results could be used to inform lifelong learning in the form of discussions between physicians with relatively long careers and those with relatively short careers, taking into account whether physicians are practicing in hospital wards or providing home medical care. To enhance the quality of care for older patients with multimorbidity, there is a need for clinical education and training that takes these factors into consideration.

## Supporting information

S1 Table Comparison of mean scores for each factor of each scale and characteristics of respondents. (XLSX)

S1 Fig Box-plot diagram of factors with statistically significant differences in the group comparison of mean scores. (TIF)

S1 Data Minimal anonymized data set necessary to replicate our study findings. (XLSX)

S1 Appendix (DOCX)
