# Supplementary figures and images for: Relationship between the characteristics of Japanese physicians involved in medical care for older adults and their approaches to treating older patients with multimorbidity
Source: PLoS One. 2024 Jun 12;19(6):e0302532. doi: 10.1371/journal.pone.0302532 (PMC11168666; doi:10.1371/journal.pone.0302532)

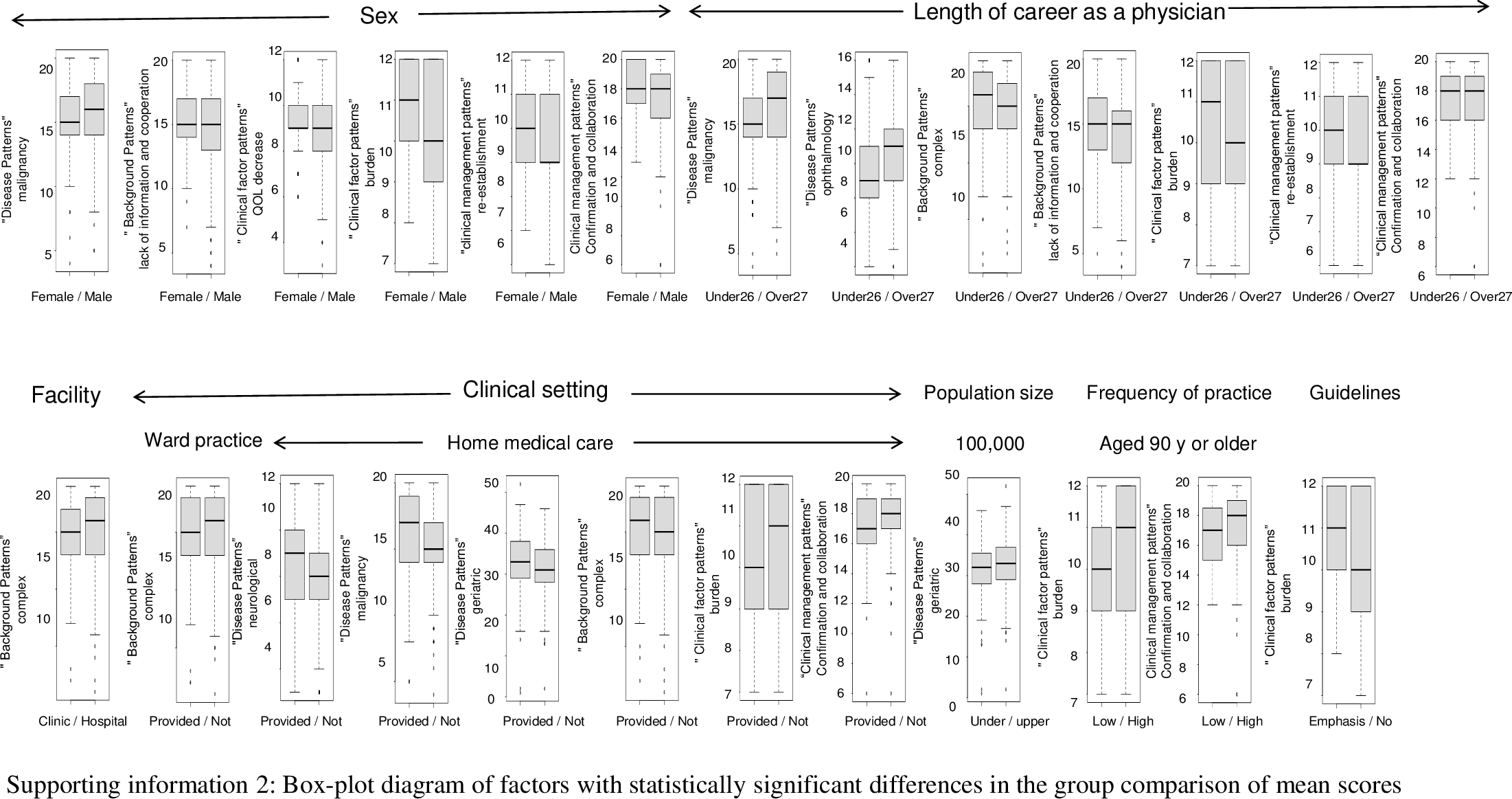

Supplement: S1 Fig — (TIF) [file pone.0302532.s002.tif]
