# Supplementary material for: Relationship between the characteristics of Japanese physicians involved in medical care for older adults and their approaches to treating older patients with multimorbidity
Source: PLoS One. 2024 Jun 12;19(6):e0302532. doi: 10.1371/journal.pone.0302532 (PMC11168666; doi:10.1371/journal.pone.0302532)
Supplement: S1 Appendix — (DOCX) [file pone.0302532.s004.docx]

APPENDICES

Appendix 1: Diseases that cause difficulties when treating older patients with multimorbidity.

1. Congestive heart failure
2. Hypertension
3. Atrial fibrillation
4. Ischemic heart disease (with history of acute myocardial infarction or hospitalization for catheterization and treatment)
5. Peripheral arterial disease (including intermittent claudication, post-bypass, gangrene, or untreated thoraco–abdominal aortic aneurysm [>5 cm])
6. Diabetes mellitus (severe: any of the three major complications or a history of hospitalization for diabetic ketoacidosis or diabetic coma)
7. Diabetes mellitus (mild: without any of the three major complications but excluding diet alone)
8. Dyslipidemia
9. Gout/hyperuricemia
10. Thyroid disease
11. Chronic lung disease (dyspnea with light exertion)
12. Chronic kidney disease (CKD grade 5 [glomerular filtration rate <15], creatinine ≥3 mg/dL, on dialysis, post-renal transplant, or uremia)
13. Cerebrovascular disease (mild sequelae, history of cerebrovascular disease without sequelae, history of transient ischemic attack)
14. Hemiplegia (including paraplegia, even if not caused by cerebrovascular disorder)
15. Neurological intractable disease
16. Peptic ulcer
17. Inflammatory bowel disease
18. Constipation
19. Collagen disease
20. Liver disease (mild liver cirrhosis without portal hypertension, chronic hepatitis)
21. Moderate to severe hepatic dysfunction (liver cirrhosis with portal hypertension)
22. Solid tumor (no apparent metastasis in the past 5 years)
23. Cancer metastasis/metastatic solid cancer
24. Lymphoma (including lymphosarcoma, macroglobulinemia, myeloma)
25. Leukemia or true erythrocytosis
26. Depression
27. Dementia
28. Sleep disorders
29. Benign prostatic hyperplasia
30. Vertigo
31. Hearing loss
32. Low back pain
33. Epilepsy
34. Spinal canal stenosis
35. Osteoporosis
36. Osteoarthritis
37. Visual impairment
38. Glaucoma
39. Cataract
40. Dental problems
41. Bedsores
42. Eczema/dermatitis
43. AIDS

Appendix 2: Patient backgrounds that cause difficulties when treating older patients with multimorbidity.

1. Severe comorbidities
2. Many social problems
3. Many psychiatric/psychological problems
4. Difficulty in setting the goals and outcomes of medical treatment
5. Difficulty communicating with patients
6. Difficult to communicate with family members
7. No key person is available
8. Patient lives alone
9. Difficulty identifying the department/medical institution where the patient is receiving treatment at another clinic or hospital
10. Difficulty in collecting clinical information during outpatient visits to other clinics or hospitals
11. Difficulty in collecting clinical information when the patient is admitted to or discharged from another hospital
12. Unable to follow general practice guidelines
13. Difficulty in collaborating with specialists in organs/areas
14. Differences of opinion on goal setting with the specialist in the organ/areas

Appendix 3: Clinical factors that are important when treating older patients with multimorbidity

1. Comorbidities of the primary disease
2. Hearing loss
3. Visual impairment
4. Wheelchair activities of daily living (ADLs)
5. Bedridden ADLs
6. Cognitive impairment
7. Depression
8. Low nutrition
9. Psychiatric complications
10. Urinary incontinence
11. History of falls
12. Certified as requiring long-term care insurance
13. Poor adherence to medications
14. Polypharmacy
15. Burdened by waiting time for outpatient department
16. Burdened by visits to the doctor and outpatient department
17. Exercise therapy decreases quality of life (QOL)
18. Dietary therapy decreases QOL
19. Medication decreases QOL
20. Burden of treatment
21. Limited support in daily living
22. Decreased ability for the patient to be cared for at home
23. Repeated emergency room visits
24. Repeated hospitalization and discharge
25. Financial problems
26. Living alone
27. Absence of a physician who coordinates and decides the overall medical policy
28. Number of medical institutions attended
29. Number of coexisting diseases
30. Age
31. Estimated life expectancy
32. Frailty

Appendix 4: Clinical management that is important when treating older patients with multimorbidity

1. Refer to practice guidelines with older adults in mind

2. Review evidence on important outcomes

3. Review indications for drug therapy for primary prevention

4. Review indications for drug therapy for secondary prevention

5. Re-evaluate drugs

6. Reassess treatment strategy

7. Evaluate patient treatment burden

8. Listen to the patient's wishes and values

9. Listen to the opinions of the family

10. Ask for the opinions of other professionals

11. Ask for the opinions of people the patient trusts

12. Present options to the patient regarding treatment goals

13. Present treatment priorities to the patient

14. Consolidate physicians who will treat patients

15. Identify a physician who will determine the overall treatment plan

16. Examine the intervals between visits to organ/area specialists

17. Clarify the role of the organ/area specialist

18. Use of long-term care insurance services

19. Multidisciplinary intervention
